# Crystal structure and Hirshfeld surface analysis of 1-[6-bromo-2-(4-fluoro­phen­yl)-1,2,3,4-tetra­hydroquinolin-4-yl]pyrrolidin-2-one

**DOI:** 10.1107/S2056989024005826

**Published:** 2024-06-25

**Authors:** Anastasia A. Pronina, Alexandra G. Podrezova, Mikhail S. Grigoriev, Khudayar I. Hasanov, Nurlana D. Sadikhova, Mehmet Akkurt, Ajaya Bhattarai

**Affiliations:** a RUDN University, 6 Miklukho-Maklaya St., Moscow, 117198, Russian Federation; b Frumkin Institute of Physical Chemistry and Electrochemistry, Russian Academy of Sciences, Leninskiy prospect 31-4, Moscow 119071, Russian Federation; c Western Caspian University, Istiqlaliyyat Street 31, AZ1001, Baku, Azerbaijan; d Azerbaijan Medical University, Scientific Research Centre (SRC), A. Kasumzade St. 14. AZ 1022, Baku, Azerbaijan; e Department of Chemistry, Baku State University, Z. Xalilov Str, Az 1148 Baku, Azerbaijan; f Department of Physics, Faculty of Sciences, Erciyes University, 38039 Kayseri, Türkiye; g Department of Chemistry, M.M.A.M.C (Tribhuvan University) Biratnagar, Nepal; Universidade de Sâo Paulo, Brazil

**Keywords:** crystal structure, acyl­ation, thienyl­allyl­amines, maleic acid amide, weak inter­actions, Hirshfeld surface analysis

## Abstract

In the title compound, C_19_H_18_BrFN_2_O, the pyrrolidine ring adopts an envelope conformation. In the crystal, mol­ecules are linked by inter­molecular N—H⋯O, C—H⋯O, C—H⋯F and C—H⋯Br hydrogen bonds, forming a three-dimensional network. In addition, C—H⋯π inter­actions connect mol­ecules into ribbons along the *b*-axis direction, consolidating the mol­ecular packing. The inter­molecular inter­actions in the crystal structure were qu­anti­fied and analysed using Hirshfeld surface analysis.

## Abstract 2

In the crystal, mol­ecules are linked by inter­molecular N—H⋯O, C—H⋯O, C—H⋯F and C—H⋯Br hydrogen bonds, forming a three-dimensional network. In addition, C—H⋯π inter­actions connect mol­ecules into ribbons along the *b*-axis direction, consolidating the mol­ecular packing.

## 1. Chemical context

**Figure 1:**
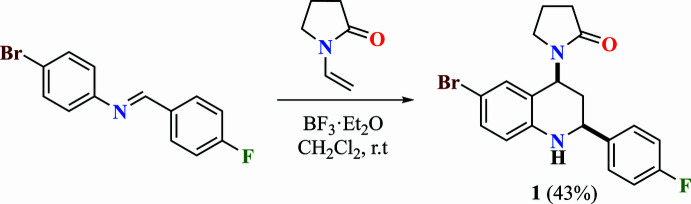
Synthesis of 1-[6-bromo-2-(4-fluoro­phen­yl)-1,2,3,4-tetra­hydro­quinolin-4-yl]pyrrolidin-2-one (I).

As a result of their presence in many plants, tetra­hydro­quinoline derivatives have long been of great inter­est to organic chemists and biochemists. The tetra­hydro­quinoline moiety can be found in many alkaloids that possess anti­malarial and anti­microbial properties (Ghashghaei *et al.*, 2018 ▸; Khalilov *et al.*, 2021 ▸; Safavora *et al.*, 2019 ▸). Various studies show that tetra­hydro­quinolines have a wide spectrum of biological activity, and some are already being used as pharmaceutical agents (Sridharan *et al.*, 2011 ▸; Akbari Afkhami *et al.*, 2017 ▸; Abdelhamid *et al.*, 2011 ▸). Modification of tetra­hydro­quinoline derivatives is effective in the search, design, and development of new drugs. However, thousands of compounds are required to find a structure that exhibits biological activity, which is why an efficient synthetic methodology for obtaining tetra­hydro­quinoline derivatives is necessary (Astudillo *et al.*, 2009 ▸; Kouznetsov *et al.*, 2004 ▸, 2007 ▸). One of the most widely used approaches for the synthesis of tetra­hydro­quinolines is the Povarov reaction, known as the aza-Diels–Alder reaction (Palacios *et al.*, 2010 ▸; Zubkov *et al.*, 2010 ▸; Zaitsev *et al.*, 2009 ▸). Herein, we have synthesized 1-[6-bromo-2-(4-fluoro­phen­yl)-1,2,3,4-tetra­hydro­quinolin-4-yl]pyrrolidin-2-one (I) by the reaction of (*E*)-*N*-(4-bromo­phen­yl)-1-(4-fluoro­phen­yl)methanimine with 1-vinyl­pyrrolidin-2-one in the presence of the most commonly used Lewis acid, diethyl ether of boron trifluoride (Fig. 1 ▸). The mild conditions and efficiency of the cyclo­addition of aromatic imines with electronically enriched alkenes make the Povarov reaction a useful tool in the synthesis of tetra­hydro­quinolines, optimization of the search for potential drugs, and obtaining hits. It should be mentioned that the conformation of the obtained 1,2,3,4-tetra­hydro­quinoline cycle plays a key role in the biological activity of a potential drug. The attached halogens (–F and –Br) as well as NH or C=O groups can participate in various sorts of inter­molecular inter­actions (Gurbanov *et al.*, 2020 ▸, 2022*a* ▸,*b* ▸; Kopylovich *et al.*, 2011*a* ▸,*b* ▸; Mahmoudi *et al.*, 2017*a* ▸,*b* ▸), which can improve the solubility of this compound. Thus, this communication is devoted to the elucidation of the spatial peculiarities of the partly hydrogenated quinoline fragment in the products of the Povarov reaction.
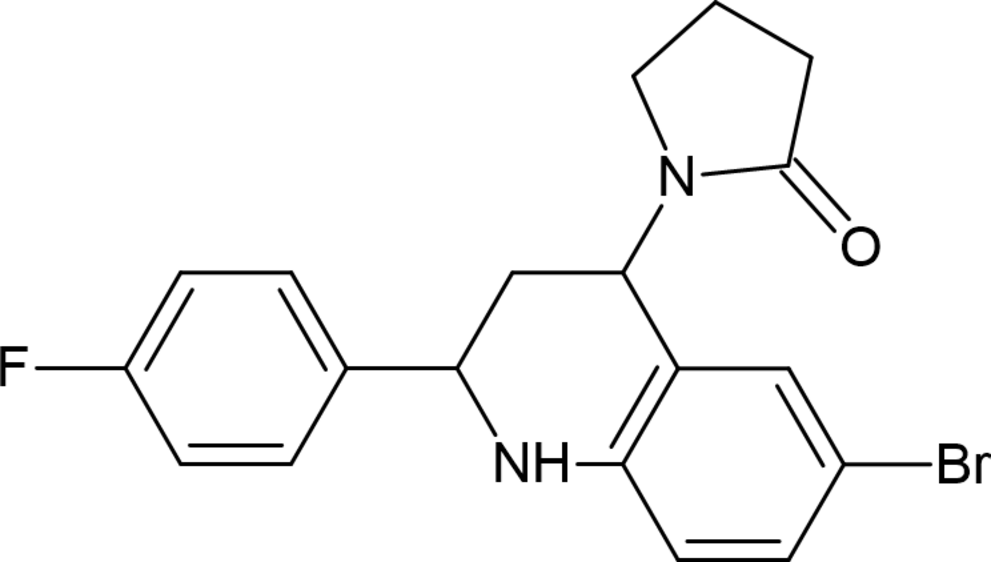


## 2. Structural commentary

**Figure 2:**
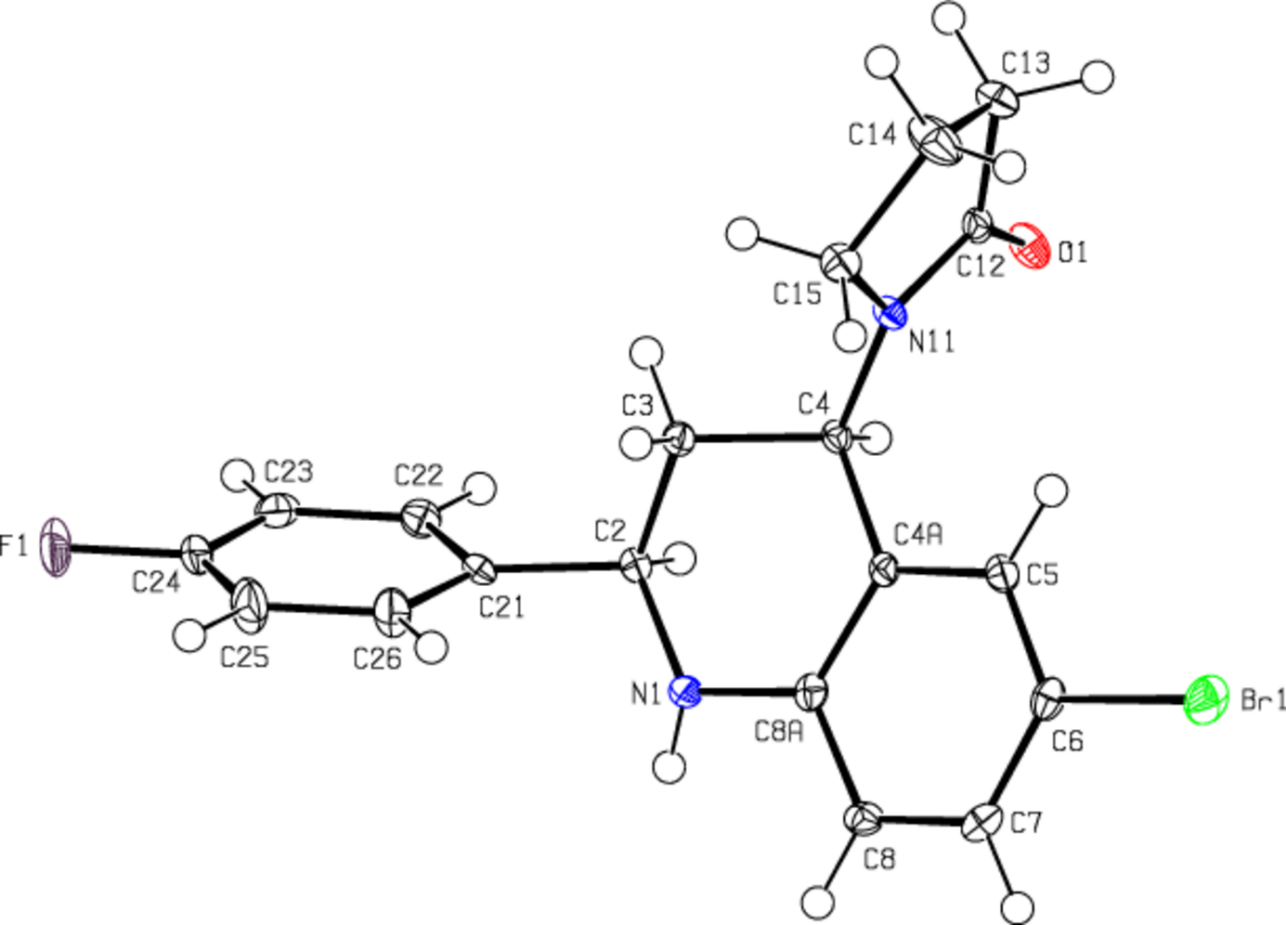
View of the title mol­ecule. Displacement ellipsoids are drawn at the 50% probability level.

In the title compound (Fig. 2 ▸), the 1,2,3,4-tetra­hydro­pyridine ring (N1/C2–C4/C4*A*/C8*A*) of the 1,2,3,4-tetra­hydro­quinoline ring system (N1/C2–C4/C4*A*/C5–C8/C8*A*) adopts an envelope conformation [the puckering parameters (Cremer & Pople, 1975 ▸) are *Q*_T_ = 0.509 (2) Å, θ = 46.3 (2)°, φ = 121.4 (3)°], while the benzene ring (C4*A*/C5–C8/C8*A*) is essentially planar (r.m.s. deviation = 0.002 Å). The plane (r.m.s deviation = 0.002 Å) of the 1,2,3,4-tetra­hydro­quinoline ring system forms angles of 65.91 (8) and 81.17 (9)°, respectively, with the fluoro­benzene ring (C21–C26) and the pyrrolidine ring (N11/C12–C15) (r.m.s deviation = 0.002 Å), which has an envelope conformation [the puckering parameters are *Q*(2) = 0.195 (2) Å, φ(2) = 107.4 (6)°]. The angle between the pyrrol­idine and fluoro­benzene rings is 71.16 (11)°. The geometric parameters in the mol­ecule are normal and in good agreement with those in the compounds discussed in the *Database survey* section.

## 3. Supra­molecular features and Hirshfeld surface analysis

**Figure 3:**
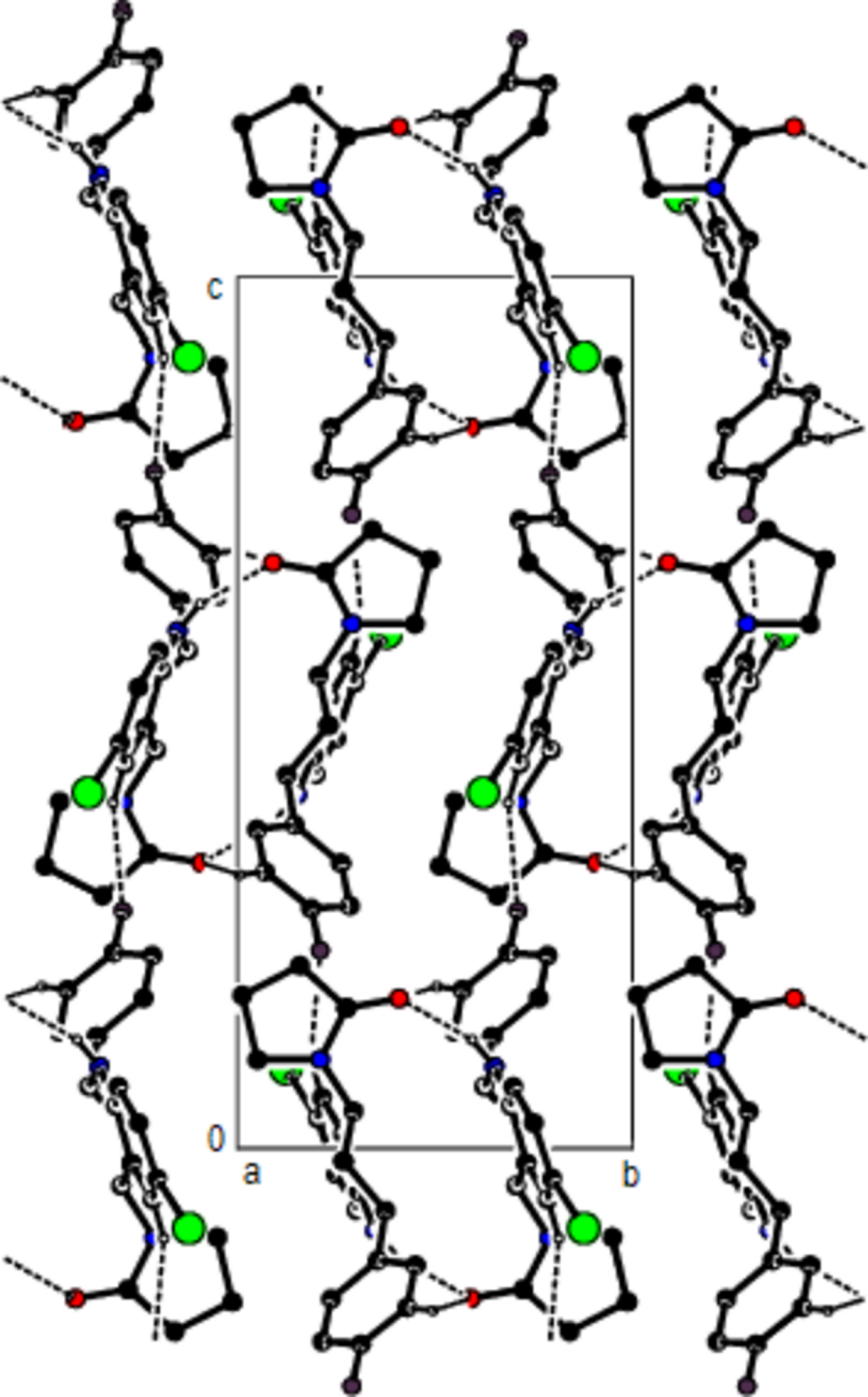
A view of the mol­ecular packing along the *a* axis of the title compound, showing the N—H⋯O, C—H⋯O, C—H⋯F and C—H⋯Br hydrogen bonds.

**Figure 6:**
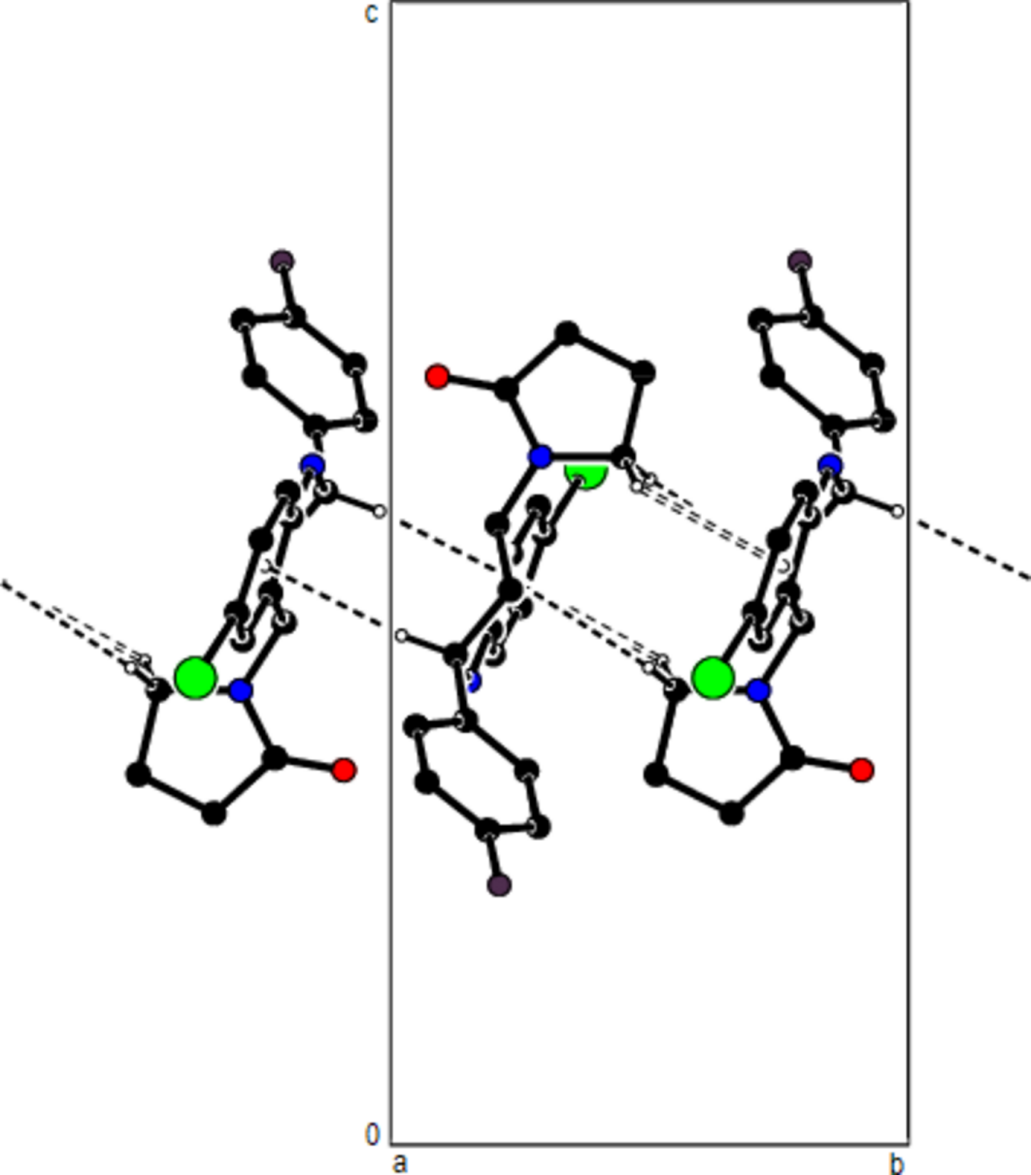
A view of the mol­ecular packing along the *a* axis of the title compound, showing the C—H⋯π inter­actions.

In the crystal, mol­ecules are linlked by inter­molecular N—H⋯O, C—H⋯O, C—H⋯F and C—H⋯Br hydrogen bonds, forming a three-dimensional network (Table 1 ▸; Figs. 3 ▸, 4 ▸ and 5 ▸). In addition, C—H⋯π inter­actions connect mol­ecules, forming ribbons along the *b*-axis direction and consol­idating mol­ecular packing (Table 1 ▸; Figs. 6 ▸, 7 ▸ and 8 ▸).

**Figure 9:**
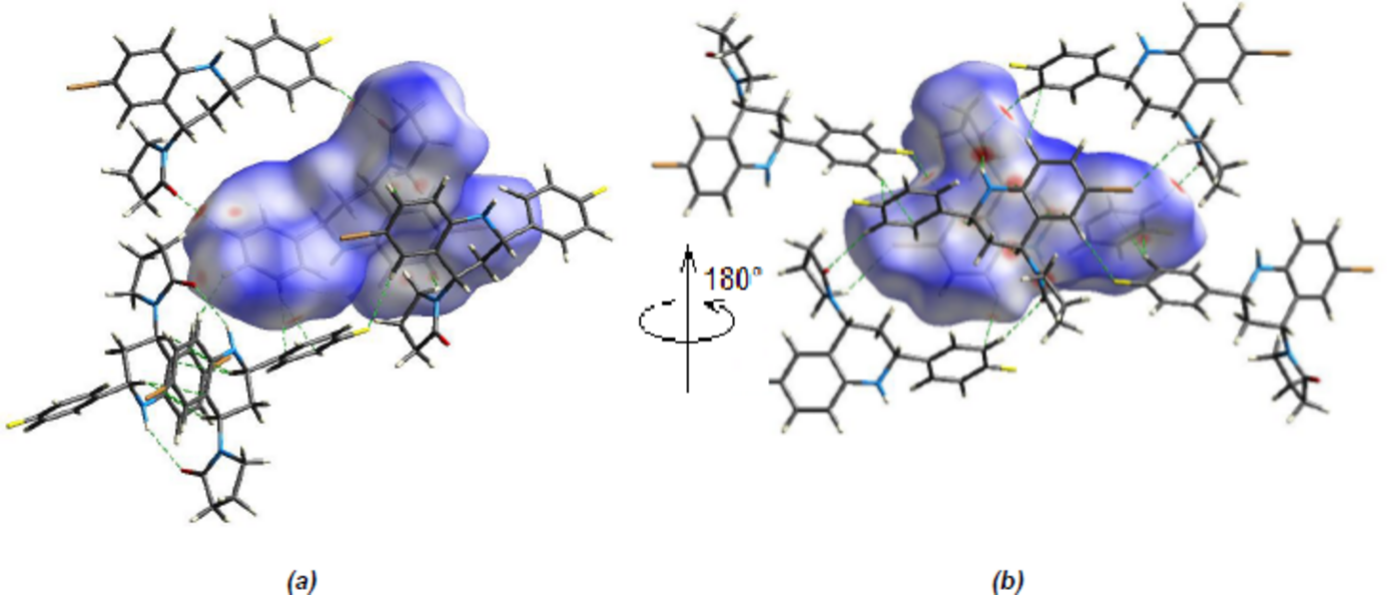
(*a*) Front and (*b*) back views of the three-dimensional Hirshfeld surface for the title compound. Some N—H⋯O, C—H⋯O and C—H⋯F inter­actions are shown as dashed lines.

To qu­antify the inter­mol­ecular inter­actions in the crystal, the Hirshfeld surfaces of the title mol­ecule and the two-dimensional fingerprints were generated with *CrystalExplorer17.5* (Spackman *et al.*, 2021 ▸). The *d*_norm_ mappings for the title compound were performed in the range −0.2398 (red) to +1.3617 (blue) a.u. On the *d*_norm_ surfaces, bright-red spots show the locations of the N—H⋯O, C—H⋯O and C—H⋯F inter­actions (Table 1 ▸; Fig. 9 ▸*a,b*).

**Figure 10:**
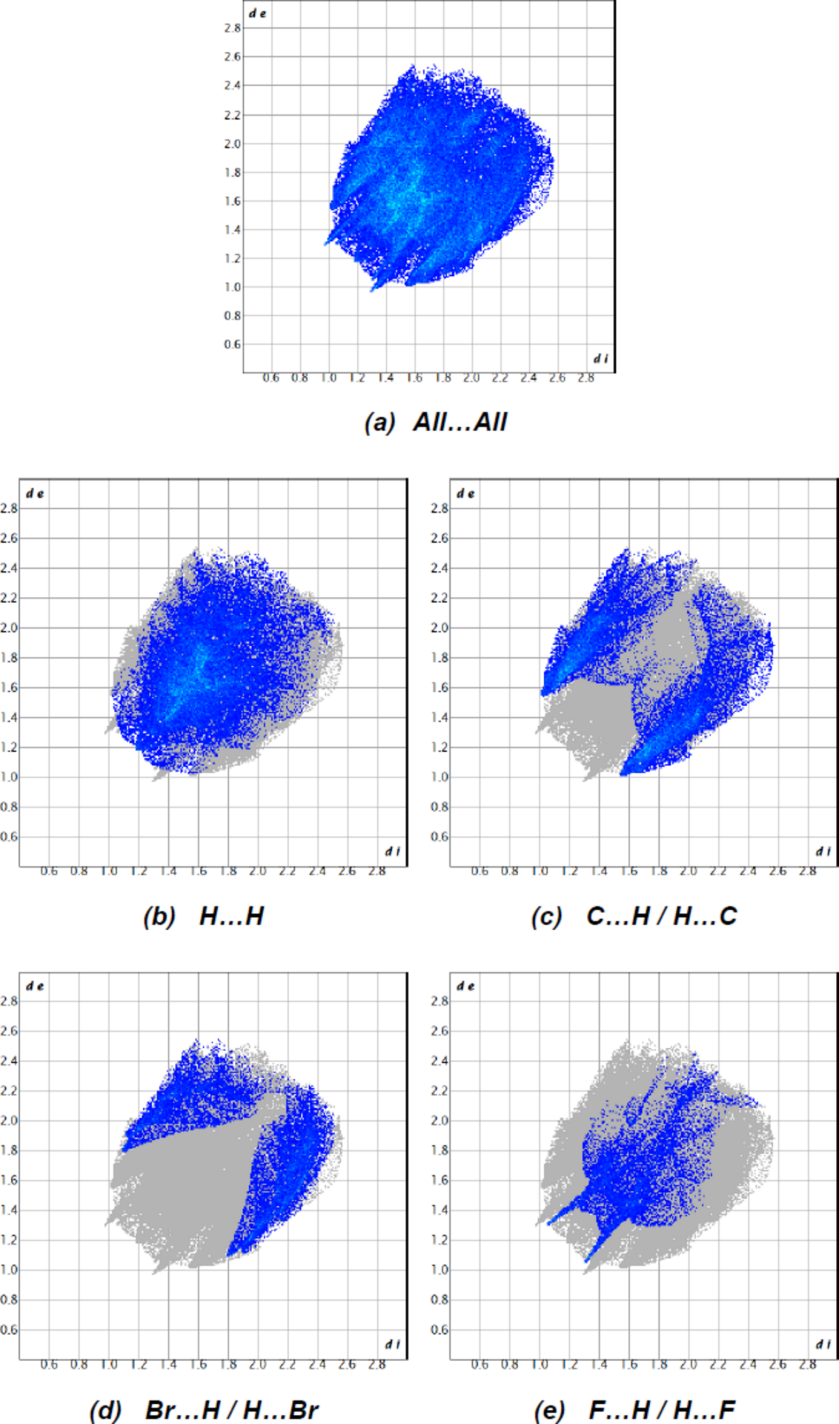
The two-dimensional fingerprint plots for the title compound showing (*a*) all inter­actions, and delineated into (*b*) H⋯H, (*c*) C⋯H/H⋯C, (*d*) Br⋯H/H⋯Br and (*e*) F⋯H/H⋯F inter­actions. The *d_i_* and *d_e_* values are the closest inter­nal and external distances (in Å) from given points on the Hirshfeld surface.

The overall two-dimensional fingerprint plot for the title compound and those delineated into H⋯H (Fig. 10 ▸*b*; 38.7%), C⋯H/H⋯C (Fig. 10 ▸*c*; 24.3%), Br⋯H/H⋯Br (Fig. 10 ▸*d*; 14.9%) and F⋯H/H⋯F (Fig. 10 ▸*e*; 9.6%) contacts are shown in Fig. 10 ▸. O⋯H/H⋯O (8.8%), Br⋯C/C⋯Br (1.8%), F⋯O/O⋯F (0.6%), F⋯C/C⋯F (0.6%), N⋯H/H⋯N (0.5%), Br⋯N/N⋯Br (0.1%) and Br⋯Br (0.1%) contacts have little directional influence on the mol­ecular packing.

## 4. Database survey

A search of the Cambridge Structural Database (CSD, Version 5.42, update of September 2021; Groom *et al.*, 2016 ▸) for similar structures with the 1,2,3,4-tetra­hydro­quinoline unit showed that the six most closely related species to the title compound are those with refcodes WACWOO (Çelik *et al.*, 2010*a* ▸), CEDNUW (Çelik *et al.*, 2010*b* ▸), SUFDEE (Jeyaseelan, *et al.*, 2015*c* ▸), NOVGAI (Jeyaseelan *et al.*, 2015*a* ▸), WUFBEG (Jeyaseelan *et al.*, 2015*b* ▸) and EZOMIR (Çelik *et al.*, 2016 ▸).

The crystal structure of WACWOO is consolidated by weak aromatic π–π inter­actions [centroid–centroid distance = 3.802 (4) Å] between the pyridine and benzene rings of the quinoline ring systems of adjacent mol­ecules. In the crystal of CEDNUW, π–π stacking inter­actions are present between the pyridine and benzene rings of adjacent mol­ecules [centroid–centroid distances = 3.634 (4) Å], and short Br⋯Br contacts [3.4443 (13) Å] occur. In the crystal of SUFDEE, mol­ecules are linked by weak C—H⋯O hydrogen bonds, generating *C*(8) and *C*(4) chains propagating along [100] and [010], respectively, which together generate (001) sheets. In the crystal of NOVGAI, inversion dimers linked by pairs of C—H⋯O hydrogen bonds generate 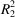
(8) loops. In the crystal of WUFBEG, inversion dimers linked by pairs of C—H⋯O hydrogen bonds generate 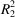
(10) loops. Additional inter­molecular C—H⋯O hydrogen bonds generate *C*(7) chains along [100]. In the crystal of EZOMIR, inversion dimers linked by pairs of N—H⋯N hydrogen bonds generate 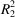
(12) loops.

## 5. Synthesis and crystallization

***N*****-[(*****E*****)-(4-Fluoro­phen­yl)methyl­idene]-4-bromaniline:** Anhydrous MgSO_4_ (3.61 g, 0.030 mol) and 4-fluoro­benzaldehyde (1.86 g, 0.015 mol) were successively added to a solution of 4-bromaniline (2.60 g, 0.015 mol) in CH_2_Cl_2_ (35 mL). After 24 h at room temperature, the reaction mixture was filtered through a silica gel layer (2 × 3 cm), eluent – CH_2_Cl_2_ (2 × 25 mL). The solvent was evaporated under reduced pressure and the residue was recrystallized from hexa­ne/EtOAc. Azomethine was obtained as a light-yellow powder in a yield of 92% (3.88 g).

**1-[6-Bromo-2-(4-fluoro­phen­yl)-1,2,3,4-tetra­hydro­quinolin-4-yl]pyrrolidin-2-one (1):** Boron trifluoride ether (0.33 mL, 0.0026 mol) and *N*-vinyl­pyrrolidone (1.50 mL, 0.014 mol) were added to a cooled solution (275–277 K) of the previously obtained azomethine (3.50 g, 0.013 mol) in freshly distilled CH_2_Cl_2_ (30 mL). After that, the suspension was mixed at room temperature for 24 h and treated with a small amount of water (0.2–0.3 mL) to decompose the catalyst. The reaction mixture was filtered through a layer of silica gel (2 × 3 cm), washed with CH_2_Cl_2_ (2 × 6 mL) and the solvent was evapor­ated under reduced pressure. The obtained product was recrystallized from a mixture of hexa­ne/EtOAc. A white microcrystalline precipitate of the title compound was isolated in a yield of 43% (2.17 g), m.p. 460.3–462.3 K. IR (KBr), ν (cm^−1^): 3344 (NH), 2956 (Ph), 2897 (Ph), 1666 (N—C=O).^1^H NMR (700 MHz, CDCl_3_, 298 K) (*J*, Hz): δ 2.00–2.10 (*m*, 4H, H-3 + H-4-pyrrole), 2.43–2.47 (*m*, 1H, H-3-pyrrole-A), 2.52–2.57 (*m*, 1H, H-3-pyrrole-B), 3.19–3.26 (*m*, 2H, H-5-pyrrole), 4.56 (*dd*, *J* = 9.5, *J* = 5.0, 1H, H-2), 5.65–5.68 (*m*, 1H, H-4), 6.48 (*d*, *J* = 8.3, 1H, H-8), 6.95 (*br.s*, 1H, H-5), 7.05–7.08 (*m*, 2H, H-2,6–C_6_H_4_–F), 7.14 (*dd*, *J* = 8.6, *J* = 2.4, 1H, H-7), 7.38–7.40 (*m*, 2H, H-3,5–C_6_H_4_–F) ppm. ^13^C NMR{^1^H} (176 MHz, CDCl_3_, 298 K) (*J*, Hz): δ 18.18, 31.19, 34.82, 42.21, 48.07, 55.68, 110.10, 115.65 (*d*, 2C, ^2^*J*_C,F_ = 21.6), 116.67, 121.05, 128.06 (*d*, 2C, ^3^*J*_C,F_ = 8.1), 129.14, 131.10, 138.15 (*br.s*, 1C), 144.59, 162.38 (*d*, ^1^*J*_C,F_ = 245.8), 175.84 ppm. ^19^F NMR{^1^H} (659 MHz, CDCl_3_, 298 K): δ −113.93 (*s*, 1F) ppm. Elemental analysis calculated (%) for C_19_H_18_BrFN_2_O: C, 58.62; H, 4.66; N, 7.20; found: C, 58.73; H, 4.53; N, 7.15. Single crystals (colourless prisms) of the title compound were grown from a mixture of hexane and ethyl acetate (∼3:1).

## 6. Refinement

Crystal data, data collection and structure refinement details are summarized in Table 2 ▸. The C-bound H atoms were placed in calculated positions (0.95–1.00 Å) and refined as riding with *U*_iso_(H) = 1.2*U*_eq_(C). The N-bound H atom was located in a difference map and freely refined.

## Supplementary Material

Crystal structure: contains datablock(s) I. DOI: 10.1107/S2056989024005826/ex2084sup1.cif

Structure factors: contains datablock(s) I. DOI: 10.1107/S2056989024005826/ex2084Isup2.hkl

Supporting information file. DOI: 10.1107/S2056989024005826/ex2084Isup3.cml

CCDC reference: 2362911

Additional supporting information:  crystallographic information; 3D view; checkCIF report

## Figures and Tables

**Figure 4:**
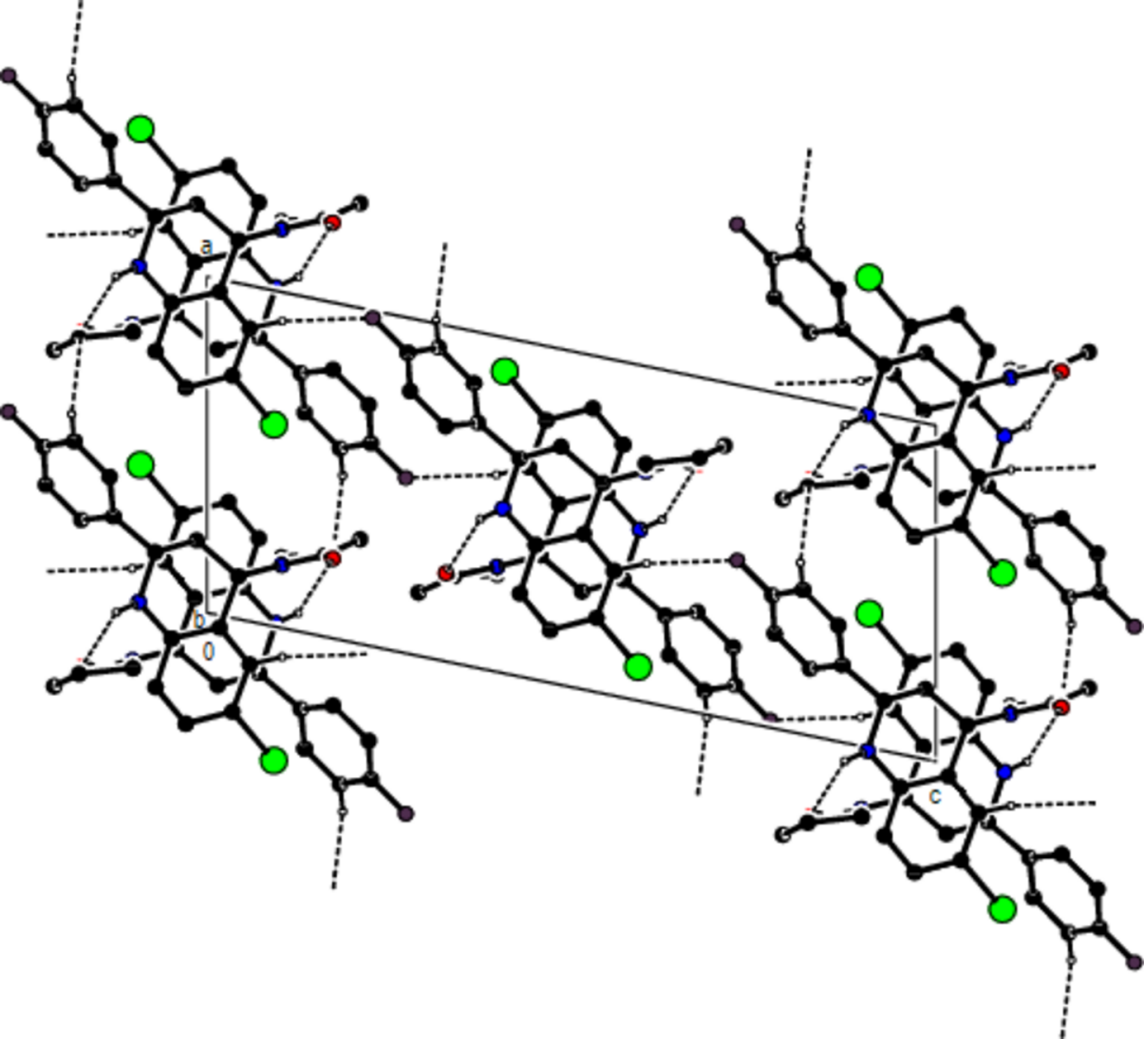
A view of the mol­ecular packing along the *b* axis of the title compound.

**Figure 5:**
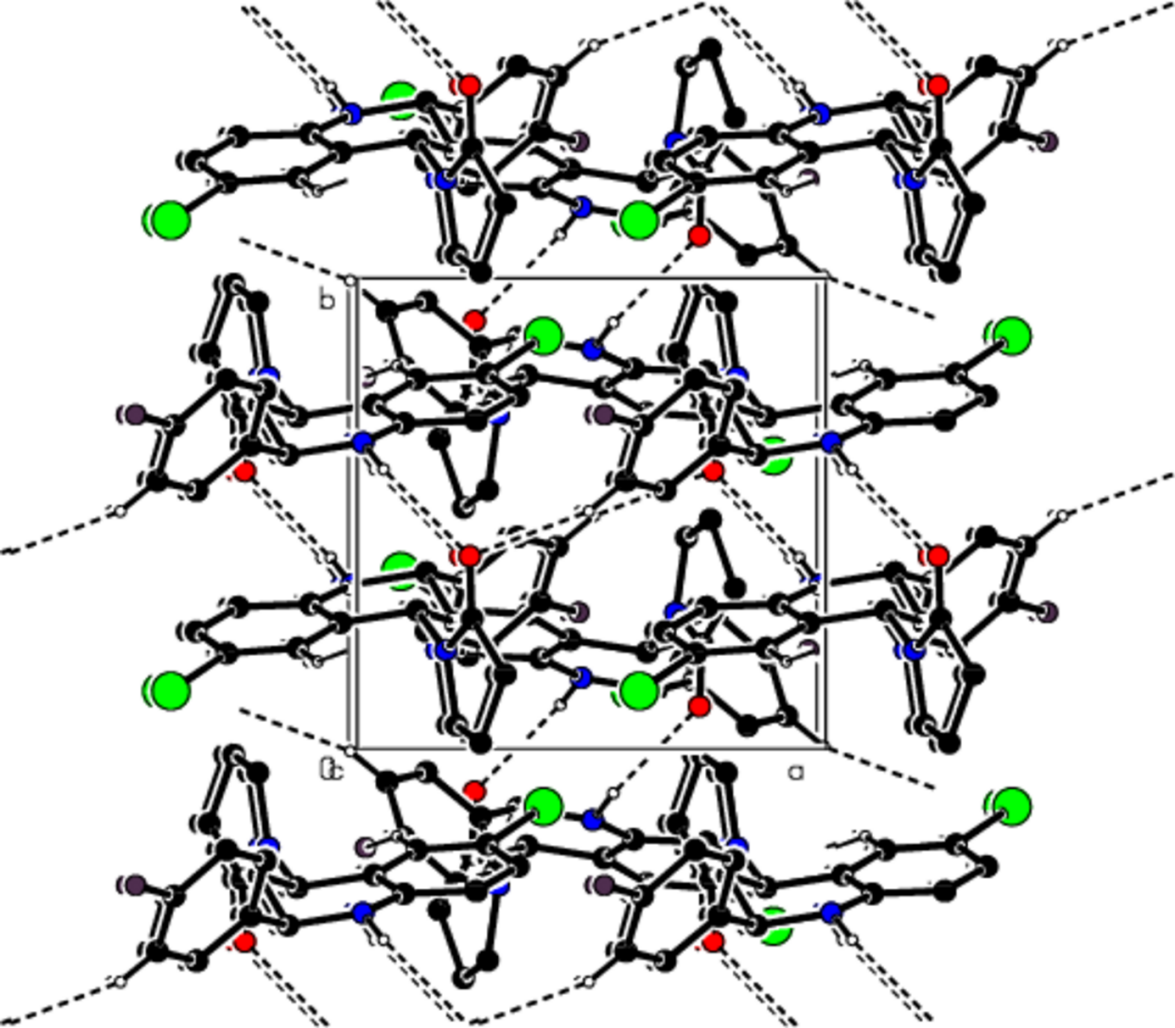
A view of the mol­ecular packing along the *c* axis of the title compound.

**Figure 7:**
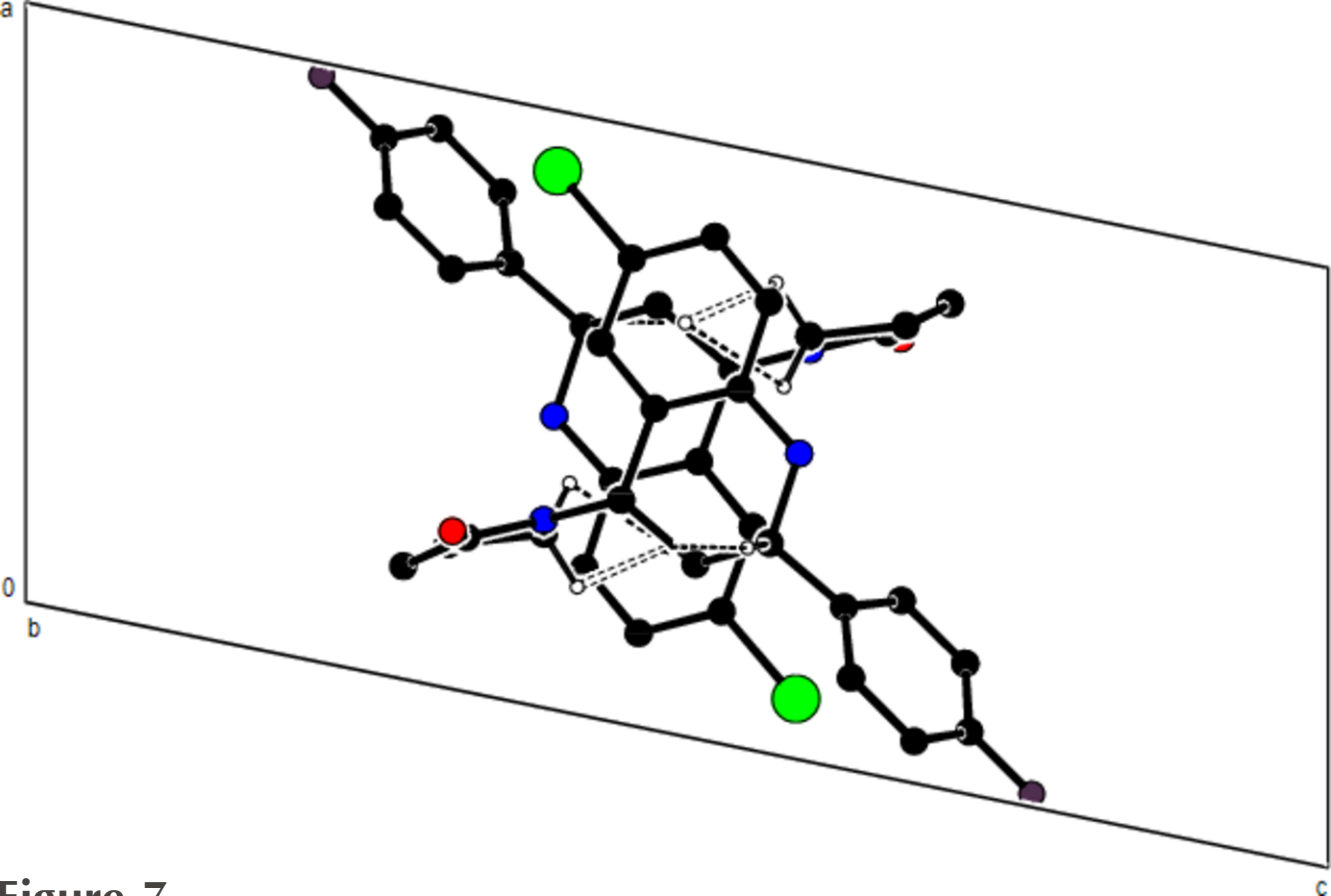
A view of the mol­ecular packing along the *b* axis.

**Figure 8:**
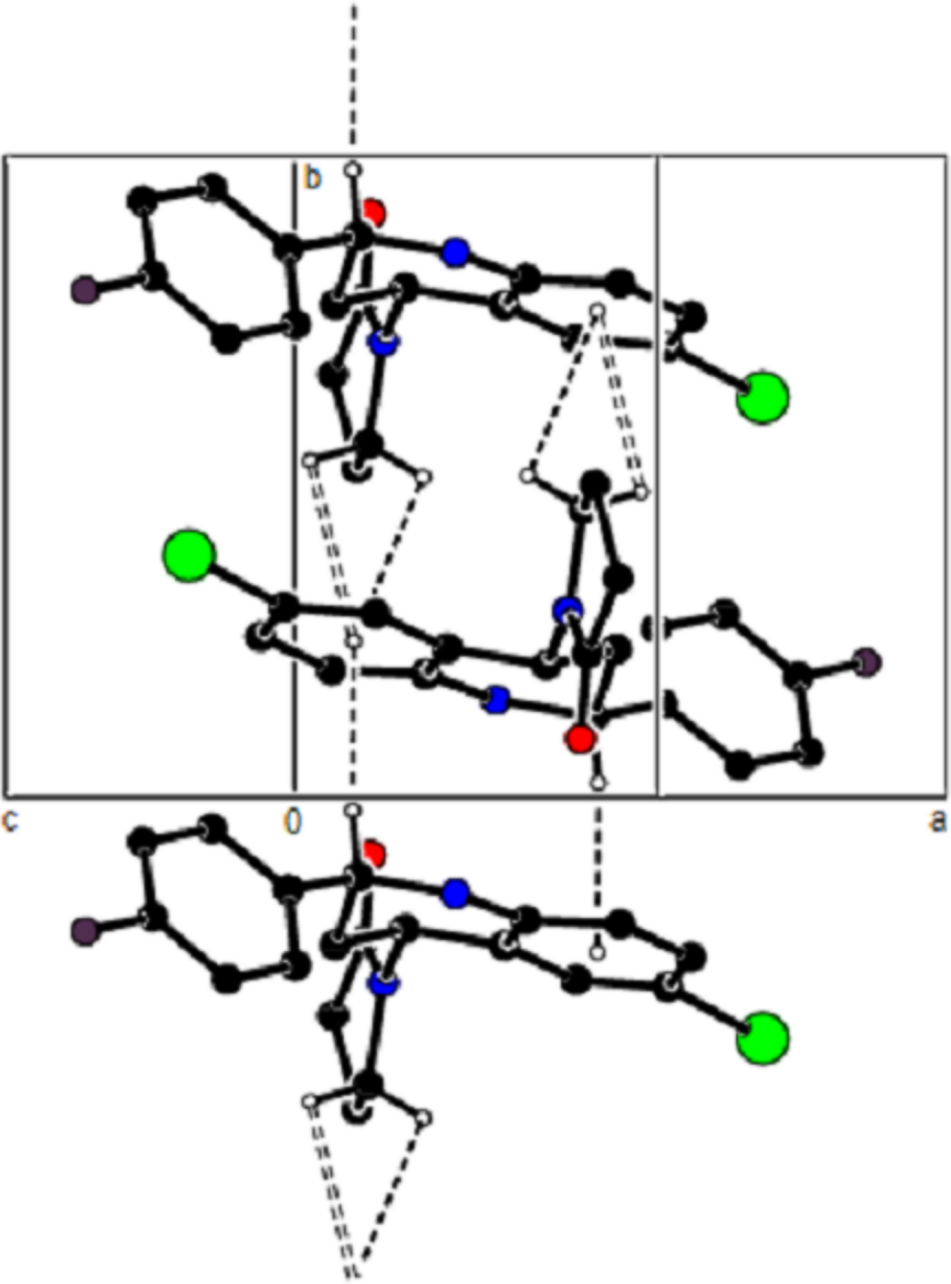
A view of the mol­ecular packing of the title compound showing supramolecular ribbons running along the *c*-axis direction.

**Table 1:** Hydrogen-bond geometry (Å, °) *Cg*3 is the centroid of the C4*A*/C5–C8/C8*A* ring.

*D*—H⋯*A*	*D*—H	H⋯*A*	*D*⋯*A*	*D*—H⋯*A*
N1—H1⋯O1^i^	0.85 (2)	2.43 (2)	3.217 (2)	154 (2)
C5—H5*A*⋯F1^ii^	0.95	2.50	3.393 (2)	157
C23—H23*A*⋯O1^iii^	0.95	2.46	3.365 (2)	159
C15—H15*B*⋯Br1^iv^	0.99	2.98	3.703 (2)	131
C2—H2*A*⋯*Cg*3^i^	1.00	2.68	3.676 (2)	172
C15—H15*A*⋯*Cg*3^v^	0.99	2.94	3.386 (2)	109
C15—H15*B*⋯*Cg*3^v^	0.99	2.97	3.386 (2)	106

Symmetry codes: (i) 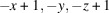; (ii) 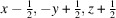; (iii) 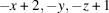; (iv) 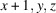; (v) 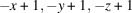.

**Table 2:** Experimental details

Crystal data	
Chemical formula	C_19_H_18_BrFN_2_O
*M*_r_	389.26
Crystal system, space group	Monoclinic, *P*2_1_/*n*
Temperature (K)	100
*a*, *b*, *c* (Å)	9.2092 (6), 9.0576 (6), 20.4085 (13)
β (°)	101.518 (2)
*V* (Å^3^)	1668.06 (19)
*Z*	4
Radiation type	Mo *K*α
μ (mm^−1^)	2.48
Crystal size (mm)	0.40 × 0.36 × 0.34

Data collection	
Diffractometer	Bruker Kappa APEXII area-detector diffractometer
Absorption correction	Multi-scan (*SADABS*; Krause *et al.*, 2015 ▸)
*T*_min_, *T*_max_	0.752, 1.000
No. of measured, independent and observed [*I* > 2σ(*I*)] reflections	28015, 4882, 3713
*R*_int_	0.057
(sin θ/λ)_max_ (Å^−1^)	0.706

Refinement	
*R*[*F*^2^ > 2σ(*F*^2^)], *wR*(*F*^2^), *S*	0.035, 0.072, 1.02
No. of reflections	4882
No. of parameters	221
H-atom treatment	H atoms treated by a mixture of independent and constrained refinement
Δρ_max_, Δρ_min_ (e Å^−3^)	0.45, −0.41

Computer programs: *APEX3* and *SAINT* (Bruker, 2018 ▸), *SHELXT2014/5* (Sheldrick, 2015*a* ▸), *SHELXL2018/3* (Sheldrick, 2015*b* ▸), *ORTEP-3 for Windows* (Farrugia, 2012 ▸) and *PLATON* (Spek, 2020 ▸).

## supplementary crystallographic information

### 1-[6-Bromo-2-(4-fluorophenyl)-1,2,3,4-tetrahydroquinolin-4-yl]pyrrolidin-2-one Crystal data

**Table:**

C_19_H_18_BrFN_2_O	*F*(000) = 792
*M**_r_* = 389.26	*D*_x_ = 1.550 Mg m^−^^3^
Monoclinic, *P*2_1_/*n*	Mo *K*α radiation, λ = 0.71073 Å
*a* = 9.2092 (6) Å	Cell parameters from 5298 reflections
*b* = 9.0576 (6) Å	θ = 3.0–27.1°
*c* = 20.4085 (13) Å	µ = 2.48 mm^−^^1^
β = 101.518 (2)°	*T* = 100 K
*V* = 1668.06 (19) Å^3^	Bulk, colourless
*Z* = 4	0.40 × 0.36 × 0.34 mm

### 1-[6-Bromo-2-(4-fluorophenyl)-1,2,3,4-tetrahydroquinolin-4-yl]pyrrolidin-2-one Data collection

**Table:**

Bruker Kappa APEXII area-detector diffractometer	3713 reflections with *I* > 2σ(*I*)
φ and ω scans	*R*_int_ = 0.057
Absorption correction: multi-scan (SADABS; Krause *et al.*, 2015)	θ_max_ = 30.1°, θ_min_ = 3.8°
*T*_min_ = 0.752, *T*_max_ = 1.000	*h* = −12→12
28015 measured reflections	*k* = −12→12
4882 independent reflections	*l* = −28→28

### 1-[6-Bromo-2-(4-fluorophenyl)-1,2,3,4-tetrahydroquinolin-4-yl]pyrrolidin-2-one Refinement

**Table:**

Refinement on *F*^2^	Primary atom site location: structure-invariant direct methods
Least-squares matrix: full	Hydrogen site location: mixed
*R*[*F*^2^ > 2σ(*F*^2^)] = 0.035	H atoms treated by a mixture of independent and constrained refinement
*wR*(*F*^2^) = 0.072	*w* = 1/[σ^2^(*F*_o_^2^) + (0.0267*P*)^2^ + 0.8068*P*] where *P* = (*F*_o_^2^ + 2*F*_c_^2^)/3
*S* = 1.02	(Δ/σ)_max_ = 0.001
4882 reflections	Δρ_max_ = 0.45 e Å^−^^3^
221 parameters	Δρ_min_ = −0.40 e Å^−^^3^
0 restraints	

### 1-[6-Bromo-2-(4-fluorophenyl)-1,2,3,4-tetrahydroquinolin-4-yl]pyrrolidin-2-one Special details

**Table:**

Geometry. All esds (except the esd in the dihedral angle between two l.s. planes) are estimated using the full covariance matrix. The cell esds are taken into account individually in the estimation of esds in distances, angles and torsion angles; correlations between esds in cell parameters are only used when they are defined by crystal symmetry. An approximate (isotropic) treatment of cell esds is used for estimating esds involving l.s. planes.

### 1-[6-Bromo-2-(4-fluorophenyl)-1,2,3,4-tetrahydroquinolin-4-yl]pyrrolidin-2-one Fractional atomic coordinates and isotropic or equivalent isotropic displacement parameters (Å^2^)

**Table:**

	*x*	*y*	*z*	*U*_iso_*/*U*_eq_	
C2	0.64897 (19)	0.1250 (2)	0.42863 (9)	0.0113 (3)	
H2A	0.664204	0.021487	0.445624	0.014*	
C3	0.7094 (2)	0.2311 (2)	0.48563 (9)	0.0114 (4)	
H3A	0.695266	0.334338	0.469667	0.014*	
H3B	0.816813	0.213924	0.501577	0.014*	
C4	0.62722 (19)	0.2058 (2)	0.54273 (9)	0.0098 (3)	
H4A	0.639259	0.099050	0.555148	0.012*	
C4A	0.46344 (19)	0.23196 (19)	0.51703 (9)	0.0097 (3)	
C5	0.3722 (2)	0.2855 (2)	0.55836 (9)	0.0125 (4)	
H5A	0.413562	0.314242	0.602937	0.015*	
C6	0.2210 (2)	0.2968 (2)	0.53445 (10)	0.0132 (4)	
C7	0.1569 (2)	0.2503 (2)	0.47072 (10)	0.0144 (4)	
H7A	0.052477	0.253929	0.455694	0.017*	
C8	0.2472 (2)	0.1984 (2)	0.42916 (10)	0.0127 (4)	
H8A	0.204136	0.166365	0.385250	0.015*	
C8A	0.4015 (2)	0.19234 (19)	0.45099 (9)	0.0110 (4)	
C12	0.74202 (19)	0.2236 (2)	0.66142 (9)	0.0117 (4)	
C13	0.8116 (2)	0.3412 (2)	0.70995 (10)	0.0170 (4)	
H13A	0.921011	0.333787	0.718707	0.020*	
H13B	0.777820	0.331985	0.752877	0.020*	
C14	0.7601 (3)	0.4872 (2)	0.67578 (10)	0.0247 (5)	
H14A	0.677041	0.529078	0.693974	0.030*	
H14B	0.842211	0.559631	0.682281	0.030*	
C15	0.7101 (2)	0.4493 (2)	0.60191 (10)	0.0146 (4)	
H15A	0.617049	0.501351	0.582257	0.018*	
H15B	0.787199	0.475484	0.576387	0.018*	
C21	0.7300 (2)	0.1449 (2)	0.37154 (9)	0.0113 (4)	
C22	0.8453 (2)	0.0493 (2)	0.36616 (10)	0.0144 (4)	
H22A	0.866503	−0.031520	0.396188	0.017*	
C23	0.9300 (2)	0.0700 (2)	0.31764 (10)	0.0169 (4)	
H23A	1.008962	0.004965	0.314036	0.020*	
C24	0.8955 (2)	0.1873 (3)	0.27535 (10)	0.0195 (4)	
C25	0.7829 (2)	0.2849 (3)	0.27849 (11)	0.0251 (5)	
H25A	0.762472	0.365242	0.248101	0.030*	
C26	0.6999 (2)	0.2627 (2)	0.32732 (10)	0.0184 (4)	
H26A	0.621590	0.328838	0.330588	0.022*	
Br1	0.10063 (2)	0.37706 (2)	0.59152 (2)	0.02069 (7)	
F1	0.97779 (13)	0.20988 (16)	0.22741 (6)	0.0311 (3)	
N1	0.48956 (17)	0.15098 (18)	0.40598 (8)	0.0127 (3)	
N11	0.68720 (16)	0.28987 (17)	0.60227 (7)	0.0101 (3)	
O1	0.73676 (15)	0.09059 (14)	0.67215 (7)	0.0159 (3)	
H1	0.446 (3)	0.094 (3)	0.3753 (12)	0.022 (6)*	

### 1-[6-Bromo-2-(4-fluorophenyl)-1,2,3,4-tetrahydroquinolin-4-yl]pyrrolidin-2-one Atomic displacement parameters (Å^2^)

**Table:**

	*U*^11^	*U*^22^	*U*^33^	*U*^12^	*U*^13^	*U*^23^
C2	0.0122 (8)	0.0126 (9)	0.0094 (9)	−0.0001 (7)	0.0027 (7)	0.0007 (7)
C3	0.0121 (8)	0.0117 (9)	0.0106 (9)	−0.0017 (7)	0.0029 (7)	−0.0004 (7)
C4	0.0124 (8)	0.0090 (9)	0.0084 (9)	−0.0013 (7)	0.0027 (7)	−0.0007 (7)
C4A	0.0128 (8)	0.0071 (8)	0.0094 (9)	−0.0022 (7)	0.0029 (7)	0.0016 (7)
C5	0.0159 (9)	0.0114 (9)	0.0106 (9)	−0.0022 (7)	0.0037 (7)	−0.0005 (7)
C6	0.0132 (9)	0.0111 (9)	0.0171 (10)	0.0006 (7)	0.0072 (8)	−0.0006 (7)
C7	0.0104 (8)	0.0125 (9)	0.0203 (10)	−0.0008 (7)	0.0030 (8)	0.0011 (8)
C8	0.0137 (9)	0.0105 (9)	0.0131 (9)	−0.0029 (7)	0.0007 (7)	−0.0011 (7)
C8A	0.0136 (9)	0.0069 (9)	0.0129 (9)	−0.0016 (7)	0.0039 (7)	0.0011 (7)
C12	0.0094 (8)	0.0164 (10)	0.0102 (9)	0.0024 (7)	0.0038 (7)	0.0011 (7)
C13	0.0177 (10)	0.0207 (11)	0.0113 (10)	−0.0011 (8)	−0.0001 (8)	−0.0019 (8)
C14	0.0411 (13)	0.0161 (11)	0.0148 (11)	−0.0047 (9)	0.0005 (10)	−0.0031 (8)
C15	0.0183 (9)	0.0106 (9)	0.0147 (10)	−0.0024 (7)	0.0027 (8)	−0.0012 (7)
C21	0.0126 (8)	0.0136 (10)	0.0071 (8)	−0.0010 (7)	0.0008 (7)	−0.0021 (7)
C22	0.0153 (9)	0.0138 (10)	0.0140 (10)	0.0004 (7)	0.0025 (8)	−0.0012 (8)
C23	0.0116 (9)	0.0220 (11)	0.0167 (10)	−0.0004 (8)	0.0016 (8)	−0.0069 (8)
C24	0.0140 (9)	0.0356 (12)	0.0103 (10)	−0.0018 (9)	0.0053 (8)	−0.0017 (9)
C25	0.0218 (11)	0.0363 (13)	0.0188 (11)	0.0066 (9)	0.0080 (9)	0.0138 (10)
C26	0.0162 (9)	0.0233 (11)	0.0171 (10)	0.0070 (8)	0.0064 (8)	0.0074 (8)
Br1	0.01630 (10)	0.02401 (11)	0.02408 (12)	0.00192 (9)	0.00963 (8)	−0.00610 (10)
F1	0.0234 (7)	0.0555 (9)	0.0186 (7)	0.0011 (6)	0.0141 (5)	0.0060 (6)
N1	0.0116 (7)	0.0173 (9)	0.0096 (8)	−0.0032 (6)	0.0026 (6)	−0.0037 (6)
N11	0.0137 (7)	0.0084 (7)	0.0079 (7)	−0.0002 (6)	0.0013 (6)	−0.0002 (6)
O1	0.0228 (7)	0.0126 (7)	0.0118 (7)	0.0042 (5)	0.0025 (6)	0.0031 (5)

### 1-[6-Bromo-2-(4-fluorophenyl)-1,2,3,4-tetrahydroquinolin-4-yl]pyrrolidin-2-one Geometric parameters (Å, º)

**Table:**

C2—N1	1.467 (2)	C12—C13	1.508 (3)
C2—C21	1.514 (2)	C13—C14	1.525 (3)
C2—C3	1.526 (2)	C13—H13A	0.9900
C2—H2A	1.0000	C13—H13B	0.9900
C3—C4	1.528 (2)	C14—C15	1.525 (3)
C3—H3A	0.9900	C14—H14A	0.9900
C3—H3B	0.9900	C14—H14B	0.9900
C4—N11	1.447 (2)	C15—N11	1.459 (2)
C4—C4A	1.513 (2)	C15—H15A	0.9900
C4—H4A	1.0000	C15—H15B	0.9900
C4A—C5	1.391 (2)	C21—C26	1.389 (3)
C4A—C8A	1.401 (3)	C21—C22	1.391 (3)
C5—C6	1.384 (3)	C22—C23	1.390 (3)
C5—H5A	0.9500	C22—H22A	0.9500
C6—C7	1.382 (3)	C23—C24	1.365 (3)
C6—Br1	1.9053 (18)	C23—H23A	0.9500
C7—C8	1.383 (3)	C24—F1	1.367 (2)
C7—H7A	0.9500	C24—C25	1.374 (3)
C8—C8A	1.403 (3)	C25—C26	1.386 (3)
C8—H8A	0.9500	C25—H25A	0.9500
C8A—N1	1.393 (2)	C26—H26A	0.9500
C12—O1	1.227 (2)	N1—H1	0.85 (2)
C12—N11	1.352 (2)		
			
N1—C2—C21	110.74 (14)	C14—C13—H13A	110.7
N1—C2—C3	109.20 (15)	C12—C13—H13B	110.7
C21—C2—C3	110.55 (14)	C14—C13—H13B	110.7
N1—C2—H2A	108.8	H13A—C13—H13B	108.8
C21—C2—H2A	108.8	C15—C14—C13	105.17 (17)
C3—C2—H2A	108.8	C15—C14—H14A	110.7
C2—C3—C4	109.03 (14)	C13—C14—H14A	110.7
C2—C3—H3A	109.9	C15—C14—H14B	110.7
C4—C3—H3A	109.9	C13—C14—H14B	110.7
C2—C3—H3B	109.9	H14A—C14—H14B	108.8
C4—C3—H3B	109.9	N11—C15—C14	103.49 (16)
H3A—C3—H3B	108.3	N11—C15—H15A	111.1
N11—C4—C4A	113.30 (14)	C14—C15—H15A	111.1
N11—C4—C3	113.41 (14)	N11—C15—H15B	111.1
C4A—C4—C3	108.86 (15)	C14—C15—H15B	111.1
N11—C4—H4A	107.0	H15A—C15—H15B	109.0
C4A—C4—H4A	107.0	C26—C21—C22	118.86 (17)
C3—C4—H4A	107.0	C26—C21—C2	121.75 (16)
C5—C4A—C8A	119.53 (17)	C22—C21—C2	119.20 (16)
C5—C4A—C4	121.70 (16)	C23—C22—C21	121.23 (18)
C8A—C4A—C4	118.66 (15)	C23—C22—H22A	119.4
C6—C5—C4A	119.94 (18)	C21—C22—H22A	119.4
C6—C5—H5A	120.0	C24—C23—C22	117.51 (18)
C4A—C5—H5A	120.0	C24—C23—H23A	121.2
C7—C6—C5	121.30 (17)	C22—C23—H23A	121.2
C7—C6—Br1	120.02 (14)	C23—C24—F1	118.44 (18)
C5—C6—Br1	118.67 (14)	C23—C24—C25	123.63 (18)
C6—C7—C8	118.99 (17)	F1—C24—C25	117.93 (19)
C6—C7—H7A	120.5	C24—C25—C26	118.0 (2)
C8—C7—H7A	120.5	C24—C25—H25A	121.0
C7—C8—C8A	120.88 (18)	C26—C25—H25A	121.0
C7—C8—H8A	119.6	C25—C26—C21	120.77 (18)
C8A—C8—H8A	119.6	C25—C26—H26A	119.6
N1—C8A—C4A	121.61 (16)	C21—C26—H26A	119.6
N1—C8A—C8	119.18 (17)	C8A—N1—C2	120.88 (16)
C4A—C8A—C8	119.19 (16)	C8A—N1—H1	113.3 (15)
O1—C12—N11	125.02 (18)	C2—N1—H1	115.7 (15)
O1—C12—C13	127.12 (17)	C12—N11—C4	121.86 (16)
N11—C12—C13	107.85 (16)	C12—N11—C15	114.52 (16)
C12—C13—C14	105.05 (16)	C4—N11—C15	123.22 (15)
C12—C13—H13A	110.7		
			
N1—C2—C3—C4	59.01 (19)	N1—C2—C21—C22	−140.90 (18)
C21—C2—C3—C4	−178.91 (15)	C3—C2—C21—C22	97.9 (2)
C2—C3—C4—N11	173.65 (15)	C26—C21—C22—C23	0.0 (3)
C2—C3—C4—C4A	−59.24 (19)	C2—C21—C22—C23	−175.11 (17)
N11—C4—C4A—C5	−22.0 (2)	C21—C22—C23—C24	−0.2 (3)
C3—C4—C4A—C5	−149.14 (17)	C22—C23—C24—F1	179.79 (18)
N11—C4—C4A—C8A	162.03 (16)	C22—C23—C24—C25	0.2 (3)
C3—C4—C4A—C8A	34.9 (2)	C23—C24—C25—C26	0.0 (3)
C8A—C4A—C5—C6	1.1 (3)	F1—C24—C25—C26	−179.62 (19)
C4—C4A—C5—C6	−174.83 (17)	C24—C25—C26—C21	−0.2 (3)
C4A—C5—C6—C7	2.6 (3)	C22—C21—C26—C25	0.2 (3)
C4A—C5—C6—Br1	−177.82 (14)	C2—C21—C26—C25	175.16 (19)
C5—C6—C7—C8	−3.2 (3)	C4A—C8A—N1—C2	9.6 (3)
Br1—C6—C7—C8	177.20 (14)	C8—C8A—N1—C2	−172.17 (17)
C6—C7—C8—C8A	0.1 (3)	C21—C2—N1—C8A	−156.30 (16)
C5—C4A—C8A—N1	174.11 (16)	C3—C2—N1—C8A	−34.3 (2)
C4—C4A—C8A—N1	−9.8 (3)	O1—C12—N11—C4	−5.8 (3)
C5—C4A—C8A—C8	−4.1 (3)	C13—C12—N11—C4	173.15 (15)
C4—C4A—C8A—C8	171.96 (16)	O1—C12—N11—C15	−178.77 (17)
C7—C8—C8A—N1	−174.75 (17)	C13—C12—N11—C15	0.2 (2)
C7—C8—C8A—C4A	3.5 (3)	C4A—C4—N11—C12	115.73 (18)
O1—C12—C13—C14	−169.00 (18)	C3—C4—N11—C12	−119.53 (17)
N11—C12—C13—C14	12.1 (2)	C4A—C4—N11—C15	−71.9 (2)
C12—C13—C14—C15	−19.0 (2)	C3—C4—N11—C15	52.8 (2)
C13—C14—C15—N11	18.7 (2)	C14—C15—N11—C12	−12.3 (2)
N1—C2—C21—C26	44.1 (2)	C14—C15—N11—C4	174.87 (16)
C3—C2—C21—C26	−77.1 (2)		

### 1-[6-Bromo-2-(4-fluorophenyl)-1,2,3,4-tetrahydroquinolin-4-yl]pyrrolidin-2-one Hydrogen-bond geometry (Å, º)

*Cg*3 is the centroid of the C4*A*/C5–C8/C8*A* ring.

**Table:**

*D*—H···*A*	*D*—H	H···*A*	*D*···*A*	*D*—H···*A*
N1—H1···O1^i^	0.85 (2)	2.43 (2)	3.217 (2)	154 (2)
C5—H5*A*···F1^ii^	0.95	2.50	3.393 (2)	157
C23—H23*A*···O1^iii^	0.95	2.46	3.365 (2)	159
C15—H15*B*···Br1^iv^	0.99	2.98	3.703 (2)	131
C2—H2*A*···*Cg*3^i^	1.00	2.68	3.676 (2)	172
C15—H15*A*···*Cg*3^v^	0.99	2.94	3.386 (2)	109
C15—H15*B*···*Cg*3^v^	0.99	2.97	3.386 (2)	106

Symmetry codes: (i) −*x*+1, −*y*, −*z*+1; (ii) *x*−1/2, −*y*+1/2, *z*+1/2; (iii) −*x*+2, −*y*, −*z*+1; (iv) *x*+1, *y*, *z*; (v) −*x*+1, −*y*+1, −*z*+1.
